# Polyetheretherketone Hybrid Composites with Bioactive Nanohydroxyapatite and Multiwalled Carbon Nanotube Fillers

**DOI:** 10.3390/polym8120425

**Published:** 2016-12-08

**Authors:** Chen Liu, Kai Wang Chan, Jie Shen, Cheng Zhu Liao, Kelvin Wai Kwok Yeung, Sie Chin Tjong

**Affiliations:** 1 Department of Physics and Materials Science, City University of Hong Kong, Tat Chee Avenue, Kowloon, Hong Kong, China; cliu266-c@my.cityu.edu.hk (C.L.); kaiwchan8-c@my.cityu.edu.hk (K.W.C.); 2 Department of Orthopedics and Traumatology, Li Ka Shing Faculty of Medicine, The University of Hong Kong, Hong Kong, China; jayjayson909@gmail.com (J.S.); wkkyeung@hku.hk (K.W.K.Y.); 3 Department of Materials Science and Engineering, South University of Science and Technology of China, Shenzhen 518055, China; liaocz@sustc.edu.cn

**Keywords:** polyetheretherketone, nanohydroxyapatite, multiwalled carbon nanotube, hybrid nanocomposites, hydrophilic surface, osteoblasts, biocompatibility

## Abstract

Polyetheretherketone (PEEK) hybrid composites reinforced with inorganic nanohydroxyapatite (nHA) and multiwalled carbon nanotube (MWNT) were prepared by melt-compounding and injection molding processes. The additions of nHA and MWNT to PEEK were aimed to increase its elastic modulus, tensile strength, and biocompatibility, rendering the hybrids suitable for load-bearing implant applications. The structural behavior, mechanical property, wettability, osteoblastic cell adhesion, proliferation, differentiation, and mineralization of the PEEK/nHA-MWNT hybrids were studied. X-ray diffraction and SEM observation showed that both nHA and MWNT fillers are incorporated into the polymer matrix of PEEK-based hybrids. Tensile tests indicated that the elastic modulus of PEEK can be increased from 3.87 to 7.13 GPa by adding 15 vol % nHA and 1.88 vol % MWNT fillers. The tensile strength and elongation at break of the PEEK/(15% nHA)-(1.88% MWNT) hybrid were 64.48 MPa and 1.74%, respectively. Thus the tensile properties of this hybrid were superior to those of human cortical bones. Water contact angle measurements revealed that the PEEK/(15% nHA)-(1.88% MWNT) hybrid is hydrophilic due to the presence of nHA. Accordingly, hydrophilic PEEK/(15% nHA)-(1.88% MWNT) hybrid promoted the adhesion, proliferation, differentiation, and mineralization of murine MC3T3-E1 osteoblasts on its surface effectively on the basis of cell culture, fluorescence microscopy, MTT assay, WST-1 assay, alkaline phosphatase activity, and Alizarin red staining tests. Thus the PEEK/(15% nHA)-(1.88% MWNT) hybrid has the potential to be used for fabricating load-bearing bone implants.

## 1. Introduction

Nowadays, there is a substantial increase in the demand of bone implants due to an increase in the numbers of patients suffering from traffic injury, aging, and bone disease [1]. The technological development of biomaterials with excellent biocompatibility for bone replacements is considered of critical importance. Metallic implants such as titanium alloy, CoCrMo alloy, and 316L stainless steel have been widely used for fabricating load-bearing hip implants and bone fixation devices due to their superior mechanical strength and ductility. However, metallic implants suffer from corrosion due to the release of metal ions by interacting with body fluid containing 0.9% NaCl. Some ions (e.g., Ni^2+^, Cr^3+^ and Co^2+^) can induce allergy, inflammation and cytotoxicity [2,3,4]. In addition, chloride anions can attack passive films formed on metallic surfaces, leading to crevice and pitting corrosion [5,6]. Metallic implants also experience a stress shielding effect due to a mismatch in stiffness between the implants and host bone, resulting in a loss of bone density. In this respect, ceramics and polymers have been investigated as possible bone substitute materials. In particular, hydroxyapatite [Ca_10_(PO_4_)_6_(OH)_2_] is of particular interest due to its resemblance to the mineral component of human bones. However, hydroxyapatite (HA) cannot be used for load-bearing implants due to its brittle nature. Thus inorganic HA in the form of particulates with sizes of several micrometers (mHA) are employed as reinforcing fillers for high-density polyethylene (HDPE) [7]. The inclusion of bioactive mHA fillers (40 vol %) renders HDPE with excellent bioactivity and biocompatibility.

Polymers with good processability and light weight exhibit beneficial effects for biomedical applications [8,9]. Degradable polymers with low mechanical strength such as polylactic acid, polyglycolic acid, and their blends are typically used for bone tissue engineering applications [10,11]. By contrast, non-degradable polymer such as polyetheretherketone (PEEK) with high temperature durability, excellent radiation stability and high Young’s modulus has found clinical applications as trauma fixation devices and spinal cages [12,13]. However, PEEK is bio-inert and hydrophobic, and cannot osteo-integrate with adjacent bone tissues upon implantation [14]. The bioactivity of PEEK can be greatly enhanced either by depositing HA coating on its surface [15], or by incorporating inorganic HA whiskers [16] and mHA particulates into its matrix [17]. In the latter case, Abu Bakar et al. added large mHA particulates up to 40 vol % into PEEK through melt compounding and injection-molding. They found that the tensile modulus of PEEK/mHA composites rises with increasing mHA content. However, the tensile strength of the composites decreases markedly as the mHA content increases. This implies that the tensile strength of PEEK/mHA composites is lower than that of pure PEEK. This is due to poor interfacial bonding between the fillers and PEEK. Further, large mHA particulates often serve as stress concentrators, thereby fracturing into small fragments during tensile testing.

Recent progress in nanotechnology research has led to the development and synthesis of novel nanomaterials with unique properties. Nanomaterials generally possess higher mechanical strength and biocompatibility compared to their micron-sized counterparts. Nanohydroxyapatite (nHA) can be synthesized by solid state reaction, co-precipitation, hydrothermal method, sol-gel process, etc. [18]. Synthetic nHA enhances protein adsorption, thereby promoting osteoblast adhesion, proliferation, and differentiation [19]. Bone tissue is a biocomposite consisting of a collagen matrix and nHA. Human cortical bone possesses an elastic modulus in the range of 7–30 GPa, tensile strength of 50–150 MPa and fracture elongation of 1%–3% [20]. Therefore, polymer/nHA composites have been investigated for making load-bearing hip prostheses and bone tissue scaffolds [21,22,23,24,25,26]. The additions of nHA to polypropylene (PP) of low tensile stiffness and strength improve its tensile performance moderately. The elastic modulus of PP/nHA nanocomposites is still much lower than that of cortical bone [21]. In this respect, PEEK with higher tensile stiffness and strength than PP is a potential matrix material of biocomposites for forming load-bearing bone implants.

Ma et al. prepared PEEK/nHA nanocomposites through vibrating ball-mill mixing followed by injection molding [27]. They found that that the elastic modulus of PEEK reaches a maximum value of 4.6 GPa by adding 40 wt % (21.5 vol %) nHA. However, the modulus of this nanocomposite is much smaller than the lower limit for the modulus of cortical bone, i.e., 7 GPa. Very recently, we incorporated 4.4–21.5 vol % nHA rods to PEEK for enhancing its mechanical stiffness and biocompatibility [23]. The results showed that the tensile modulus of PEEK-based composites increases with increasing nHA content. It increases from 3.86 GPa (pure PEEK) to a maximum value of 7.85 GPa by adding 21.5 vol % (40 wt %) nHA. The elastic modulus of PEEK/(21.5 vol % nHA) nanocomposite reaches 7.85 GPa, being slightly higher than the lower bound in elastic modulus of cortical bone. However, PEEK/(21.5 vol % nHA) nanocomposite is very brittle having a fracture elongation of only 0.63%. These results reveal that the PEEK/(21.5 vol % nHA) nanocomposite is unsuitable for making load-bearing implants due to its brittleness. To address this issue, carbon nanofibers (CNFs) of 1.6–1.9 vol % are added to the PEEK/nHA composites for enhancing their stiffness. So PEEK/(15 vol % nHA)-(1.9 vol % CNF) biocomposite achieves a maximum elastic modulus of 6.54 GPa, being smaller than that of cortical bone.

As recognized, carbon nanotubes possess extraordinary high tensile modulus and strength. Multiwalled carbon nanotubes (MWNTs) possess an elastic modulus of 0.9 TPa and tensile strength of 150 GPa [28]. Thus MWNTs are effective nanofillers for biocomposites as they promote the adhesion and proliferation of osteoblasts on their surfaces [29,30]. By incorporating into polymers, MWNTs enhance the mechanical property and biocompatibility of resulting composites. Thus hybridizing inorganic nHA with MWNTs can effectively generate PEEK-based nanocomposites with good tensile stiffness and strength as well as excellent biocompatibility for biomedical load-bearing applications.

## 2. Experimental Section

### 2.1. Materials

**Figure 1:**
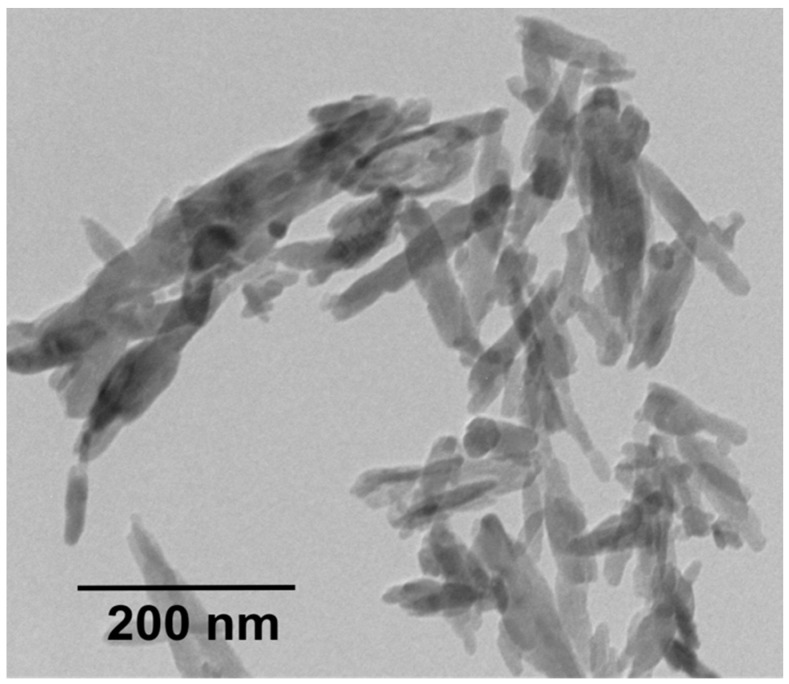
Transmission electron microscopy (TEM) image of nanohydroxyapatite (nHA).

PEEK-Optima (molecular weight 20,800 g/mol) pellets and MWNTs were bought from Invibio Company and Nanostructured & Amorphous Materials Inc. (Los Alamos, NM, USA), respectively. Nanohydroxyapatite (nHA) powders were purchased from Nanjing Emperor Nano Materials (Nanjing, China). Transmission electron microscopy (TEM; Philips CM20, Philips, Amsterdam, The Netherlands) micrograph revealed nHA exhibiting a width of 20 nm and a length of 100 nm (Figure 1). PEEK pellets and nHA were dried in an oven at 55 °C overnight prior to melt-compounding.

### 2.2. Preparation of Nanocomposites

PEEK/(15 vol % nHA)-MWNT hybrids with chemical compositions as listed in Table 1 were fabricated by melt-mixing and injection molding. For comparison, pure PEEK and binary PEEK/MWNT nanocomposites were also prepared. The compositions of binary PEEK/MWNT and ternary PEEK/(15 vol % nHA)-MWNT nanocomposite in this article were expressed in volume percentage. From our previous work [23], elastic modulus and fracture strain of the PEEK/(15 vol % nHA) nanocomposite were 6.2 GPa and 2.71%, respectively. Thus the tensile performance of this composite can be further improved by adding MWNTs. Accordingly, the nHA content of hybrid nanocomposites in this study was fixed at 15 vol %.

In the melt-mixing process, dried PEEK pellets and nHA were initially mixed in an extruder (C.W. Brabender Instruments, South Hackensack, NJ, USA) at a screw rotation speed of 30 rpm for 45 min. The mixing temperatures of Brabender from hopper to extrusion die were maintained at 360–380–390–395–380–360 °C, respectively. The extrudates were granulized by a pelletizer and loaded into Brabender again for second mixing under the same conditions. The purpose was to achieve homogeneous dispersion of nanofillers in the polymer matrix. The extruded products were pelletized again, dried overnight in an oven, and finally fed into an injection molder (Toyo TI-50H, Toyo Machinery & Metal Co., Akashi, Japan) to make dog-bone tensile bars and circular disks. The disks were mainly used for cell culture, cell viability, and alkaline phosphatase activity measurements.

### 2.3. Material Characterization

X-ray diffraction (XRD) patterns of the composite specimens were obtained using a Bruker D2 Phaser X-ray diffractometer (Karlsruhe, Germany) equipped with Cu-Kα radiation (λ = 0.154 nm) at 30 kV. The patterns were recorded from 10° to 60°. The morphologies of injection molded composite specimens were examined in a field-emission SEM (Jeol JSM 7100F; Tokyo, Japan). The composites were dipped in liquid nitrogen and then fractured by a hammer. The fractured surfaces were then coated with a thin carbon film.

Differential scanning calorimetry (DSC) tests were determined with a TA Instruments model 2910 (TA Instruments, New Castle, DE, USA) under a protective nitrogen atmosphere. The previous thermal history of the specimens was removed by an initial heating cycle of 20 °C/min from ambient to 350 °C, followed by holding for 3 min at this temperature. The specimens were then cooled at a rate of 20 °C/min and the cooling traces were recorded accordingly.

### 2.4. Tensile Tests

Tensile tests were performed at room temperature using an Instron tester (model 5567, Instron Corp., Norwood, MA, USA) at a crosshead speed of 10 mm/min. The elastic modulus of PEEK and its nanocomposites was determined from the linear region of stress-strain curves. Five samples of each composition were tested, and the average values were reported.

### 2.5. Water Contact Angle Tests

Water contact angles of nanocomposite specimens were measured using Rame Hart 500-F1 advanced goniometer equipped with DROP image software (Rame-Hart Instrument Co., Succasunna, NJ, USA). Each test run was performed by producing a water droplet and its subsequent spreading into a spherical cap on the specimen surface.

### 2.6. Cell Culture

Murine MC3T3-E1 pre-osteoblasts were cultured in Dulbecco’s Modified Eagle’s Medium (DMEM, Thermo Scientific, Pittsburgh, PA, USA) supplemented with 10% fetal bovine serum, penicillin, and streptomycin. Injection molded disks were sliced into small round shapes for cell cultivation and proliferation measurements. These samples were ground with SiC papers of different grades, followed by rinsing with 70% ethanol and phosphate buffer saline (PBS) solutions. Rinsed samples were placed in a 96-well plate. Afterwards, a cell suspension was pipetted at 10^4^ cells per well. The 96-well plate was incubated in a humidified atmosphere of 5% CO_2_/95% air at 37 °C for desired periods. The culture medium was changed every two days. Following the incubation, the samples were washed with phosphate-buffered saline (PBS) to remove unattached cells, fixed with 10% formaldehyde, dehydrated in a graded series of ethanol. Dehydrated cells were critical point dried and sputter deposited with gold in preparation for SEM examination. Moreover, micrographs of osteoblasts cultured on the samples were taken at day 1 and day 3 using a fluorescence microscope. Cells were washed with PBS and fixed with methanol-free formaldehyde for 30 min at each time point. After that, the samples were washed with PBS and stained with Alexa Fluor 488 (A12379, ThermoFisher Scientific, Carlsbad, CA, USA) at room temperature for 40 min to label F-actin filaments, and stained the nuclei with Hoechst 33342 (Sigma Aldrich, St. Louis, MO, USA) for 5 min. The samples were then washed three times and stored in PBS before imaging.

### 2.7. Cell Metabolic Activity

The cell metabolic activity was determined with 3-(4,5-dimethylthiazol-2-yl)-2,5-diphenyltetrazolium bromide (MTT), and 2-(4-iodophenyl)-3-(4-nitrophenyl)-5-(2,4-disulfophenyl)-2*H*-tetrazolium (WST-1) assays. Rinsed samples (number of samples, *n* = 5) were placed in a 96-well plate. A suspension of 10^4^ cells per well was seeded into the 96-well plate. The plate was incubated in a humidified atmosphere of 5% carbon dioxide in air at 37 °C for 3, 7 and 10 days, respectively. The culture medium was refreshed every 3 days. At days 3, 7 or 10, the medium was aspirated, then 10 μL MTT solution (5 mg MTT:1 mL DMEM) was added to each well and incubated for 4 h at 37 °C. Thus MTT reacted with mitochondrial enzyme of viable osteoblasts, producing insoluble formazan crystals. The formazan was dissolved in 10% sodium dodecyl sulfate (SDS)/0.01 M hydrochloric acid. The absorbance of dissolved formazan was measured at a wavelength of 570 nm using a multimode detector (Beckman Coulter DTX 880, Beckman Coulter Inc., Fullerton, CA, USA), with a reference wavelength of 640 nm. Those wells with MTT and DMEM were used as a negative control.

For WST-1 assay, the samples (*n* ≥ 5) were first seeded with osteoblasts at 37 °C for 3, 7 and 10 days, respectively. After cell culturing for every prescribed time period, tetrazolium salt was added to each well followed by incubation for 4 h at 37 °C to produce water soluble formazan. The amount of water-soluble formazan was quantified by the absorbance at 450 nm. The cell metabolic activity of both assays was calculated by comparing the absorbance of osteoblastic cells cultured on the nanocomposite sample to that of the cells seeded on pure PEEK. The results were expressed in terms of mean ± standard deviation (SD).

### 2.8. Cellular Differentiation

The alkaline phosphatase (ALP) activity of osteoblasts on PEEK and its nanocomposites (number of samples, *n* = 5) was measured with a colorimetric assay kit (No. 2900-500, Stanbio Laboratory, Boerne, TX, USA). The kit used colorless 4-nitrophenyl phosphate as a substrate. The enzyme ALP of the cells hydrolyzed the substrate to color 4-nitrophenol and an inorganic phosphate. In the process, test samples were placed in each well of a 48-well plate. Osteoblasts were seeded on the samples for 3, 7 and 14 days. The culture medium was changed every 3 days. At each time period, the cells were rinsed with PBS, and lysed with 0.1% triton X-100 at 4 °C for 30 min. The cell lysates were then centrifuged at 4 °C followed by pipetting 10 μL supernatant liquid of each sample in a 96-well plate. Finally, *p*-nitrophenyl phosphate was introduced into the plate. The absorbance of *p*-nitrophenol formed was measured using a spectrophotometer (Beckman Coulter DTX 880) at 405 nm. The ALP activity was normalized to the total protein level of the samples determined by the Bio-Rad protein Assay (Bio-Rad, Hercules, CA, USA).

Calcium mineralization of differentiating osteoblast on PEEK and its nanocomposites was measured with Alizarin red-S (Sigma Aldrich, Darmstadt, Germany) after cultivation with MC3T3-E1 pre-osteoblasts for 28 days. Disk samples of 10 mm diameter (number of samples, *n* = 5) were placed in a 24-well plate. At the end of the experiment, cell cultures were fixed in 4% paraformaldehyde, washed twice with PBS and then stained with Alizarin red-S. To quantify calcium mineralization, the alizarin red stained cultures were incubated with 10% cetyl-pyridinium chloride for 30 min in each well to release calcium-bound alizarin red into solution. Then extracted supernatant was transferred to a 96-well plate. The optical absorbance of the released alizarin red was measured at 562 nm using a spectrophotometer.

### 2.9. Statistical Analysis for Cellular Tests

All cell viability and differentiation assays were performed in triplicate for each treatment and the results were expressed as mean ± standard deviation (SD). A two-way ANOVA was used to determine the significance level of difference and *p* < 0.05 was considered to be statistically significant.

## 3. Results and Discussion

### 3.1. Structure and Morphology

**Figure 2:**
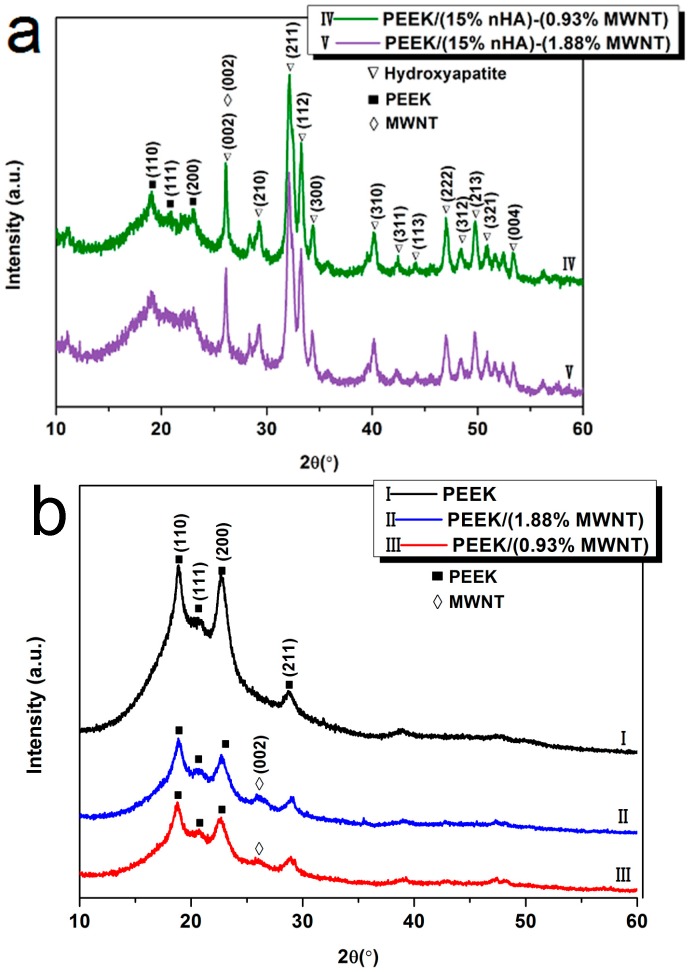
X-ray diffraction (XRD) patterns of (**a**) PEEK/(15% nHA)-(0.93% MWNT) and PEEK/(15% nHA)-(1.88% MWNT) hybrids and (**b**) pure PEEK and PEEK/MWNT nanocomposites.

Figure 2a shows the XRD patterns of PEEK/(15% nHA)-(0.93% MWNT) and PEEK/(15% nHA)-(1.88% MWNT) hybrids. For the purposes of comparison, XRD patterns of pure PEEK, PEEK/(0.93% MWNT) and PEEK/(1.83% MWNT) composites are depicted in Figure 2b. Pure PEEK exhibits characteristic peaks at around 2θ = 18.8°, 20.7°, 22.9° and 28.9°, corresponding to (110), (111), (200) and (211) crystalline planes, respectively [31]. The patterns of PEEK/MWNT nanocomposites show additional peak at 26.6° due to the (002) reflection of nanotubes. For both PEEK/(15% nHA)-(0.93% MWNT) and PEEK/(15% nHA)-(1.88% MWNT) hybrids, the peaks arising from the crystalline planes of nHA fillers can be clearly observed in addition to the PEEK reflections. Moreover, the (002) peak of nHA located at ~26.4° overlaps with the (002) peak of MWNTs.

**Figure 3:**
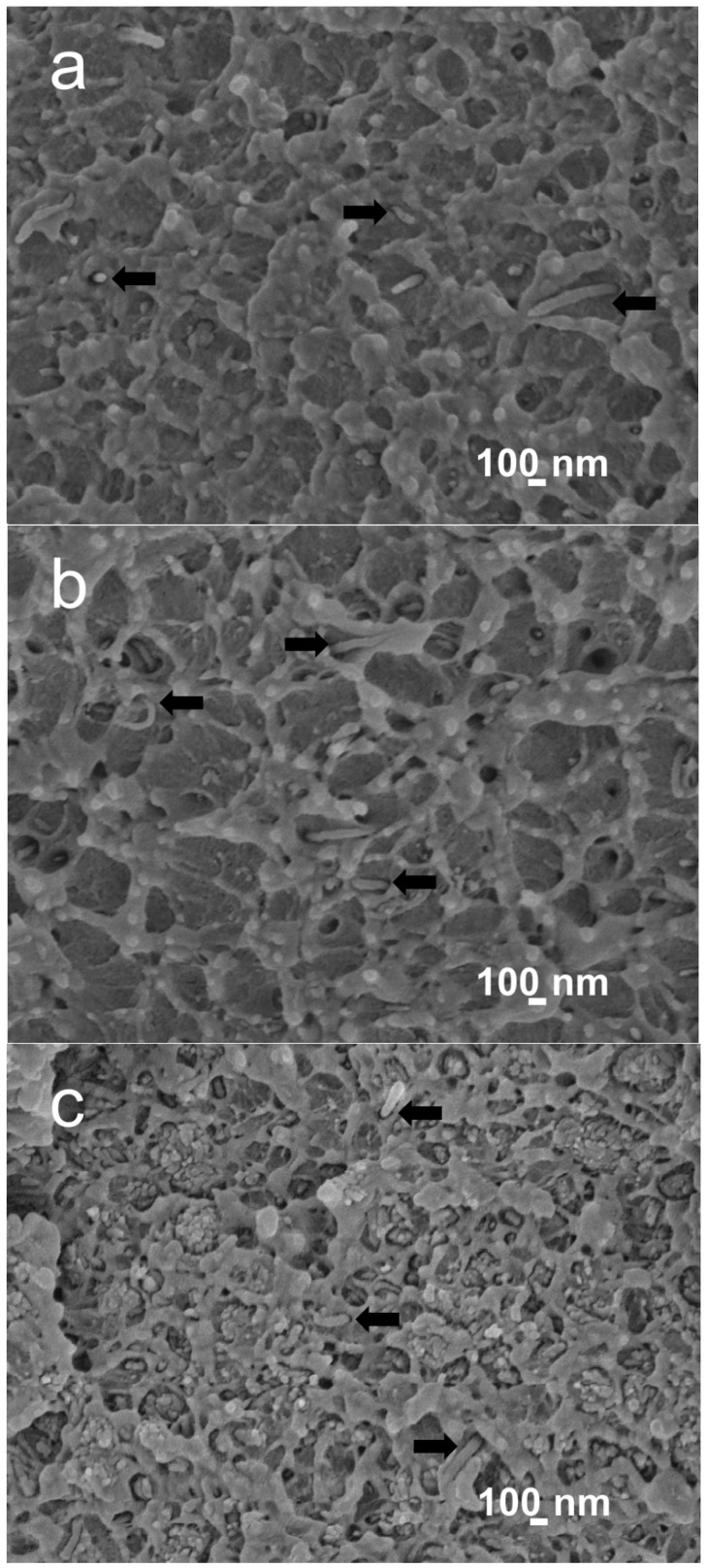
Scanning electron microscopy (SEM) images of (**a**) PEEK/(0.93 vol % MWNT); (**b**) PEEK/(1.88 vol % MWNT) and (**c**) PEEK/(15% nHA)-(1.88% MWNT) nanocomposites. Black arrows indicate MWNT fillers.

Figure 3a,b are the SEM images of PEEK/MWNT nanocomposites showing the homogeneous dispersion of MWNT fillers in the PEEK matrix. It can be seen that the PEEK matrix is quite rough, indicating the ductile nature of the nanocomposites. Figure 3c shows the SEM image of PEEK/(15% nHA)-(1.88% MWNT) hybrid. Some nHA agglomerates can be readily seen due to higher nHA filler loading in the hybrid.

### 3.2. Crystallization Behavior

**Figure 4:**
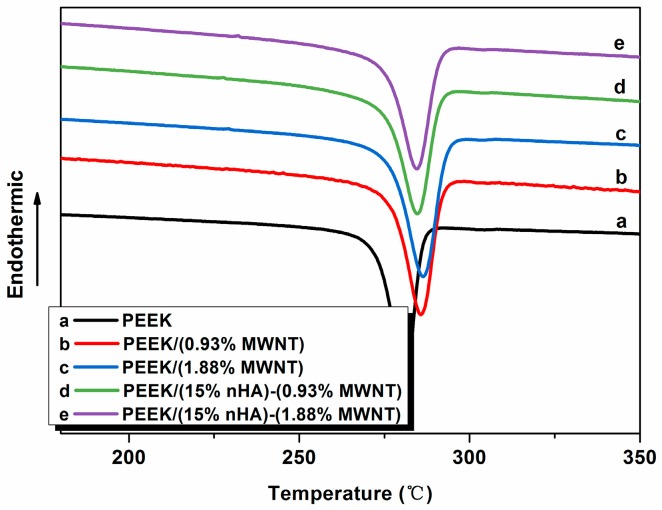
Differential scanning calorimetry (DSC) cooling curves of PEEK and its nanocomposites.

Figure 4 shows typical DSC cooling scans of neat PEEK and its nanocomposites. The crystallization temperature (*T*_c_) and the crystallization enthalpy (Δ*H*_c_) can be determined from these curves, and tabulated in Table 2. The degree of crystallinity (*X*_c_) of PEEK and its nanocomposites is calculated from the following equation:

(1) $$X_{c}\left(\%\right)=\frac{\Delta H_{c}}{\left(1-\Phi \right)\times \Delta H_{m}}\times 100\%$$

where Δ*H*_m_ is the melting enthalpy of the 100% crystalline PEEK, i.e., 130 J/g [31], and Φ is the weight fraction of the filler of the nanocomposites. The *X*_c_ values of the specimens studied are also listed in Table 2. DSC results indicate that the additions of low MWNT levels to PEEK increase its crystallization temperature. The *T*_c_ value of pure PEEK is 281.14 °C, and increases to 285.91 and 286.58 °C by adding 0.93% and 1.88% MWNTs, respectively. These demonstrate that MWNTs serve as effective nucleation sites for PEEK crystallites on cooling from melting temperature, leading to an increase of the degree of crystallinity. By adding 15% nHA to the PEEK/(0.93% MWNT) and PEEK/(1.83% MWNT), the *T*_c_ values appear to decrease. Moreover, *X*_c_ values of PEEK/(15% nHA)-(0.93% MWNT) and PEEK/(15% nHA)-(0.93% MWNT) hybrids also decrease due to the agglomeration of nHA fillers as shown in Figure 4.

Furthermore, the full width at half-maximum (FWHM) of crystallization peak of PEEK increases with increasing MWNT content. The FWHM values of PEEK and its nanocomposites are also listed in Table 2. This increase in the width of the crystallization peak of PEEK/MWNT nanocomposites due to MWNT additions could be attributed to a wider crystal size distribution [32]. However, the addition of 15% nHA to the PEEK/MWNT nanocomposites leads to a slight decrease of the FWHM of crystallization peak and a marked decrease in crystallinity. Thus a large amount of nHA fillers that agglomerate in the PEEK matrix cannot act as effective sites for polymeric chain nucleation.

### 3.3. Tensile Behavior

**Figure 5:**
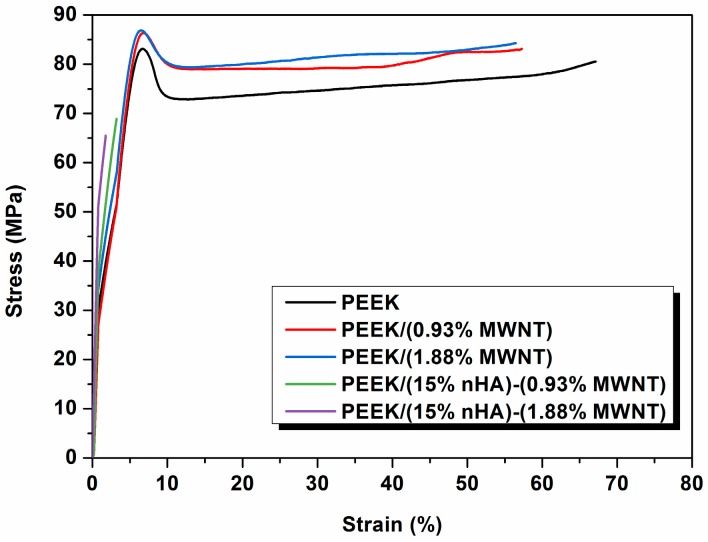
Tensile stress-strain curves of PEEK and its nanocomposites.

Figure 5 shows the stress-strain curves of PEEK and its nanocomposites. The tensile parameters of these samples and human cortical bone are summarized in Table 3. It is obvious that the elastic modulus of PEEK can be increased from 3.87 to 4.25 GPa by adding 1.88 vol % MWNTs. The elongation at break of PEEK is reduced from 67.1% to 56.48% by adding 1.88 vol % MWNTs. However, the PEEK/(15% nHA)-(1.88% MWNT) hybrid still exhibits ductile feature as evidenced by the SEM micrograph as shown in Figure 3b. MWNTs with exceptionally high stiffness also exhibit high mechanical flexibility upon the application of large deformation strains [33]. Thus the additions of low MWNT contents to PEEK enhance its tensile stiffness and strength, with a small reduction in tensile ductility. On the other hand, the addition of 15 vol % nHA to PEEK increases it stiffness to 6.2 GPa, but with a drastic reduction in fracture elongation to 2.71% due to the brittle nature of nHA [23]. From Table 3, the addition of 1.88 vol % MWNT to PEEK/(15% nHA) leads to a further increase in elastic modulus from 6.20 to 7.13 GPa. The tensile strength and elongation at break of the PEEK/(15% nHA)-(1.88% MWNT) hybrid are 64.48 MPa and 1.74%, respectively. Therefore, all the tensile properties of PEEK/(15% nHA)-(1.88% MWNT) hybrid are superior to those of human cortical bone. This demonstrates that the PEEK/(15% nHA)-(1.88% MWNT) hybrid can be used for fabricating load-bearing bone implants in terms of mechanical property consideration. From Table 2, PEEK/(15% nHA)-(0.93% MWNT) and PEEK/(15% nHA)-(1.88% MWNT) hybrids exhibit lower *X*_c_ values comparing with binary PEEK/MWNT composites. As a result, PEEK/(15% nHA)-(0.93% MWNT) and PEEK/(15% nHA)-(1.88% MWNT) hybrids exhibit lower tensile strength than binary PEEK/MWNT composites as shown in Table 3. As recognized, polymers and their composites with higher degree of crystallinity exhibit better tensile strength due to the presence of more ordered polymer crystallites.

### 3.4. Wettability

**Figure 6:**
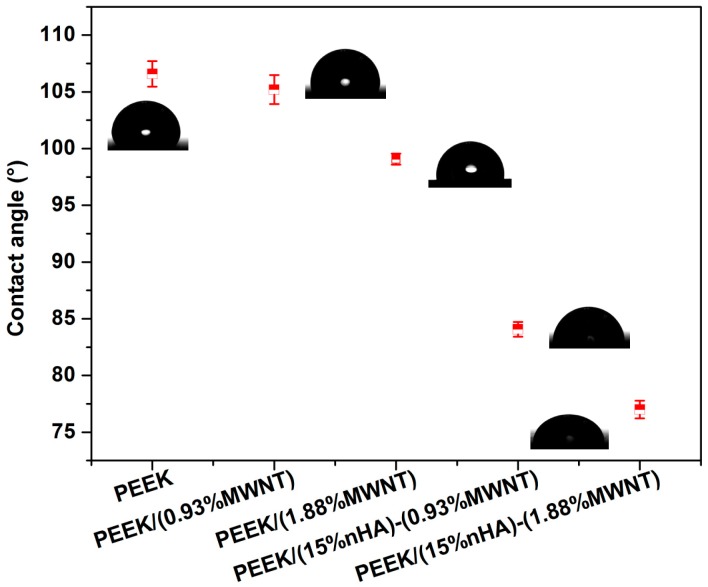
Contact angles formed by spreading of water droplets on PEEK and its nanocomposites.

Contact angle is a measure of the wettability of a solid when a liquid comes in contact with it. A hydrophobic solid surface forms when the water contact angle is larger than 90°. In this case, the water droplet does not spread but rather forms a spherical cap resting on the solid surface with a contact angle. By contrast, the hydrophilic solid surface shows a water contact angle smaller than 90°. Figure 6 shows the appearances of water droplets and contact angles on the surfaces of all samples studied. Pure PEEK displays a contact angle of 106.58° ± 1.13°, revealing its hydrophobic nature. Adding 0.93 vol % and 1.88 vol % MWNTs to PEEK decreases its water contact angles. At 1.88 vol % MWNT loading, the contact angle reduces to 99.07° ± 0.50°. However, contact angle drops markedly to 84.08° ± 0.65° and 76.99° ± 0.78° by hybridizing 15% nHA with 0.93% MWNTs and 1.88% MWNTs, respectively. This is due to the presence of nHA, which is hydrophilic in nature. Thus hydrophilic PEEK/(15% nHA)-(0.93% MWNT) and PEEK/(15% nHA)-(1.88% MWNT) hybrids favor adhesion of osteoblasts on their surfaces.

From the literature, enhancing the hydrophilicity of the polymer surface leads to increased cell spreading and adhesion. Upon implantation into the body, water molecules interact with the material surfaces followed by protein adsorption. These surface proteins play a key role in cell adhesion, proliferation, and subsequent differentiation [34,35]. The bound proteins on the material surface provide recognition sites for cell adhesion via specific cell receptors such as integrins [35,36].

### 3.5. Cellular Adhesion

**Figure 7:**
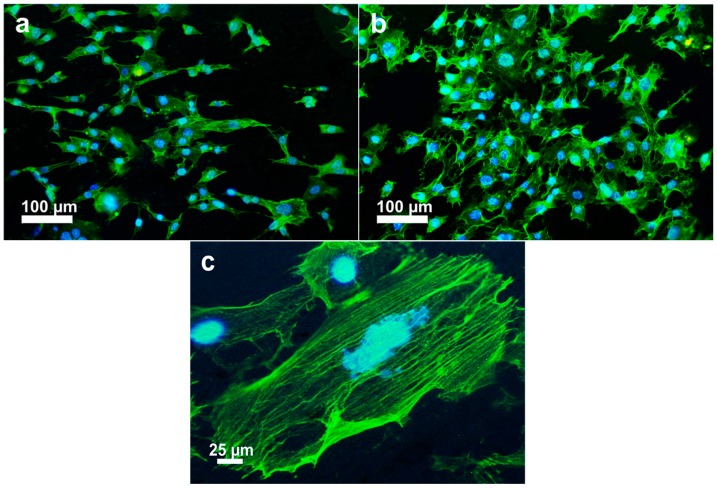
Fluorescence micrographs of osteoblasts cultured on PEEK/(15% nHA)-(0.93% MWNT) hybrid and stained for F-actin cytoskeleton (green) and nucleus (blue) for (**a**) 1 day and (**b**) 3 days; (**c**) High magnification image showing the morphology of well-organized actin filaments of a cell at day 3.

**Figure 8:**
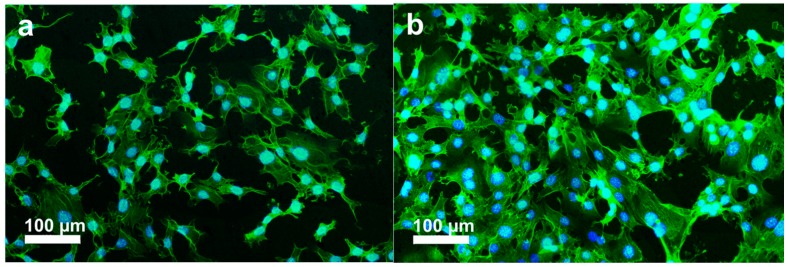
Fluorescence micrographs of osteoblasts cultured on PEEK/(15% nHA)-(1.88% MWNT) hybrid and stained for F-actin cytoskeleton (green) and nucleus (blue) at (**a**) day 1 and (**b**) day 3.

**Figure 9:**
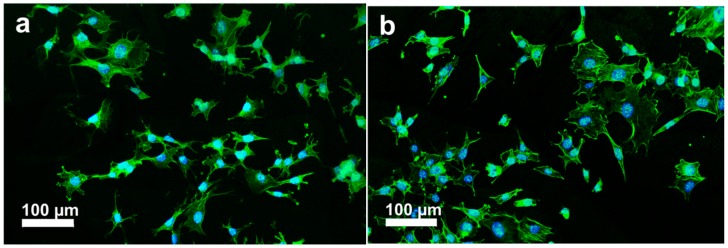
Fluorescence micrographs of osteoblasts cultured on (**a**) PEEK/(0.93% MWNT) and (**b**) PEEK/(1.88% MWNT) nanocomposites and stained for F-actin cytoskeleton (green) and nucleus (blue) for 3 days.

The initial cell-biomaterial interactions mimic to a certain extent the natural communication of cells with extracellular matrix (ECM). Cell adhesion is mediated by the integrins of cell surface receptor and the ECM proteins (e.g., fibronectin, collagen), which in turn are sensitive to the chemical composition and wettability of biomaterials [37]. Integrins are transmembrane proteins that bind ECM and interact with the cytoskeleton at focal adhesion complexes. Once integrins adhere to such molecules, they are activated and clustered into nascent adhesions [37,38]. Therefore, osteoblast adhesion on the biomaterial surface is evidenced by markedly expedited cell spreading and dense actin fiber formation. PEEK exhibits a relatively hydrophobic surface, thereby limiting cell attachment. By adding bioactive nHA and MWNTs, the resulting PEEK hybrids become hydrophilic as discussed above. Figure 7a–c shows the fluorescence micrographs of osteoblasts cultured on the PEEK/(15% nHA)-(0.93% MWNT) hybrid for 1 and 3 days, respectively. These micrographs show extensive spreading of osteoblasts and distinct actin stress fibers, especially for hybrids cultivated with osteoblasts for 3 days. Furthermore, more distinct actin stress fibers are observed on the PEEK/(15% nHA)-(1.88% MWNT) hybrid as expected (Figure 8a,b). The higher expression level of actin and focal adhesion contacts demonstrate enhanced cellular activity and stable cytoskeleton, thereby facilitating progressive development of cellular processes [39]. For comparison, fluorescence micrographs of osteoblasts cultured on the PEEK/(0.93% MWNT) and PEEK/(1.88% MWNT) nanocomposites for 3 days are shown in Figure 9a,b, respectively. These images reveal that osteoblast can attach and spread on the PEEK/MWNT composite surfaces in the absence of nHA, but with lower amounts of the cell adhesion. It is noted that these fluorescence figures provide qualitative observations only.

**Figure 10:**
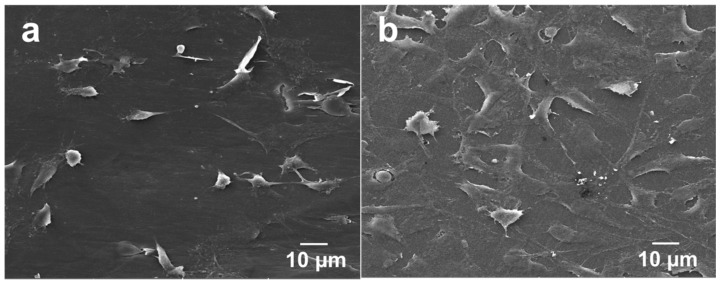
SEM images of PEEK/(1.88% MWNT) nanocomposite cultured with osteoblasts for (**a**) 2 and (**b**) 4 days.

**Figure 11:**
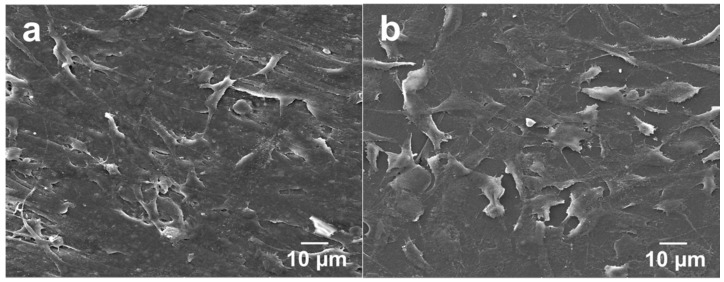
SEM images of PEEK/(15% nHA)-(0.93% MWNT) hybrid cultured with osteoblasts for (**a**) 2 and (**b**) 4 days.

The adherence and spreading of osteoblasts on a material surface can also be examined through SEM imaging. Figure 10a,b show the SEM micrographs of PEEK/(1.88% MWNT) nanocomposites seeded with osteoblasts for 2 and 4 days, respectively. Apparently, osteoblasts attach firmly on these nanocomposite surfaces after cultivation for 2 days. At day 4, they then proliferate and spread flatly on the surfaces in which many neighbor cells link with each other through cytoplasmic extension. The PEEK/(1.83% MWNT) nanocomposite surface is almost covered with osteoblasts after culturing for 4 days. It is evident that MWNTs act as effective sites for osteoblastic adhesion. Interestingly, osteoblasts attach and spread entirely on the PEEK/(15% nHA)-(0.93% MWNT) hybrid surface cultured for 2 days (Figure 11a). At day 4, the osteoblastic feature remains unchanged (Figure 11b).

### 3.6. Cellular Metabolic Activity

**Figure 12:**
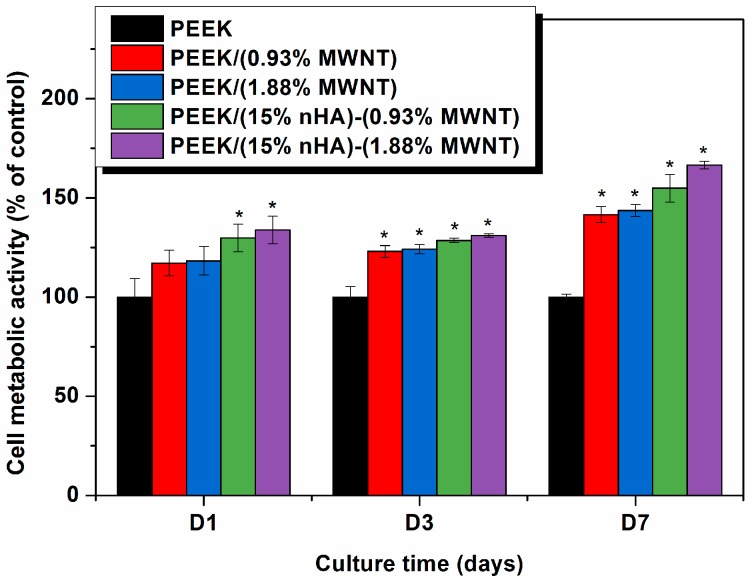
3-(4,5-Dimethylthiazol-2-yl)-2,5-diphenyltetrazolium bromide (MTT) assay results showing cell metabolic activity of murine MC3T3-E1 pre-osteoblasts grown on PEEK and its nanocomposites for 1, 3 and 7 days. * represents *p* < 0.05. Significance is assessed for each time-point assayed.

The MTT and WST-1 assays can measure viable cell number [40], while Mwenifumbo et al. demonstrated that MTT assay is related to cell metabolic activity [41]. A live/dead stain would be used to measure cell viability. Figure 12 shows the results of MTT measurements for PEEK and its nanocomposites. PEEK/(0.93% MWNT) and PEEK/(1.88% MWNT) composites exhibit higher cell metabolic activity comparing to pure PEEK specimen, especially after cultivation for 7 days. Thus MWNT fillers promote the growth of osteoblasts on the nanocomposite surfaces. It has been reported in the literature that carbon nanotubes may exhibit cytotoxic effects towards biological cells [42,43]. The cytotoxicity is mainly caused by individual carbon nanotubes dispersed in the cell culture medium or organic suspensions. As such, individual carbon nanotubes can penetrate cell membrane and reside in the cytoplasm [44,45]. On the other hand, no cytotoxic effects are observed in carbon nanotube fillers of polymer nanocomposites [21,22,23]. This is because carbon nanotube fillers are embedded firmly within the matrix of polymer nanocomposites. In this case, they serve as the active sites for the attachment of osteoblasts. From Figure 12, a marked increase in cell metabolic activity can be seen in both PEEK/(15% nHA)-(0.93% MWNT) and PEEK/(15% nHA)-(1.88% MWNT) hybrids. Thus the presence of inorganic nHA in such hybrids offers a beneficial effect in enhancing their biocompatibility. From the water contact measurements, these hybrids are hydrophilic due to the presence of nHA. On the contrary, PEEK/(0.93% MWNT) and PEEK/(1.83% MWNT) composites exhibit hydrophobic behavior because of the absence of nHA. As discussed above, increased surface hydrophilicity enhances protein adsorption and cell spreading. Thus hydrolytic materials favor the adhesion and proliferation of osteoblasts on their surfaces [34,35,36,37,38,39,40,41,42,43,44,45,46].

**Figure 13:**
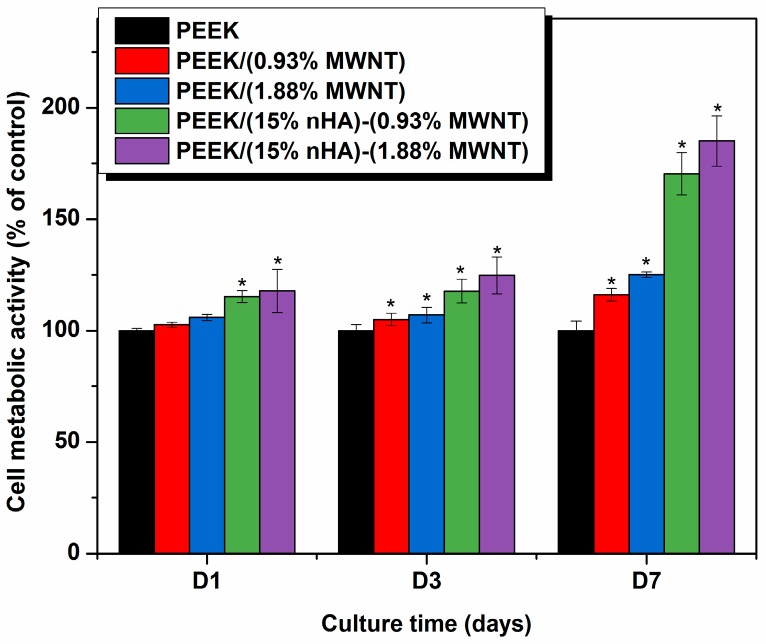
2-(4-Iodophenyl)-3-(4-nitrophenyl)-5-(2,4-disulfophenyl)-2*H*-tetrazolium (WST-1) assay results showing cell metabolic activity of murine MC3T3-E1 pre-osteoblasts grown on PEEK and its nanocomposites for 1, 3 and 7 days. * represents *p* < 0.05. Significance is assessed for each time-point assayed.

It is worth-noting that MTT measurements tend to give lower cellular metabolic activity for carbon nanotubes due to the formation of insoluble formazan products [47]. MTT assay is a colorimetric test based on the reduction of the yellowish, water-soluble tetrazolium salt into a water-insoluble, purple formazan product by mitochondrial enzyme succinate dehydrogenase of living cells in the culture. The reduction occurs only when reductase enzymes are active. Dead cells are incapable of converting MTT into formazan. MTT assay needs an additional solubilization step to dissolve color formazan crystals. The intensity of the color gives a relative count of viable cells. Such formazan crystals do not dissolve completely in an organic solvent, particularly in the presence of carbon nanotubes [47]. The undissolved crystals in a solvent give low colorimetric readings in the spectrophotometric measurements, causing low cell metabolic activity. By contrast, WST-1 assay produces water soluble formazan and does not form insoluble clusters like MTT. Therefore, WST-1 assay generates reliable results for assessing cell metabolic activity. Figure 13 shows the cell metabolic activity measured by the WST-1 assay for the PEEK-based nanocomposites. Apparently, WST-1 assay gives considerably higher cell metabolic activity than MTT assay. At day 7, the amount of viable cells for PEEK/(15% nHA)-(0.93% MWNT) and PEEK/(15% nHA)-(1.88% MWNT) hybrids is relatively high as compared to PEEK/(0.93% MWNT) and PEEK/(1.83% MWNT) composites.

For the MTT and WST assays, the amount of formazan crystals produced is directly proportional to the number of metabolically active cells in the culture. Both assays reveal that, after 7 days, cells cultured on the PEEK/(15% nHA)-(0.93% MWNT) and PEEK/(15% nHA)-(1.88% MWNT) hybrids exhibit a significantly higher metabolic activity than on the PEEK control specimen. As aforementioned, nHA enhances protein adsorption, thereby promoting osteoblast adhesion, proliferation, and differentiation [19]. In other words, proteins that can induce osteoblast adhesion, including fibronectin and vitronectin, have been reported to have higher adsorption rates on nHA [19,48]. Moreover, osteoblastic cells also attach and survive on the MWNT materials, producing higher metabolic activity on the basis of MTT results [41]. Therefore, PEEK/(15% nHA)-(0.93% MWNT) and PEEK/(15% nHA)-(1.88% MWNT) hybrid nanocomposites exhibit enhanced cell activity compared to the control PEEK specimen.

### 3.7. Cellular Differentiation and Mineralization

**Figure 14:**
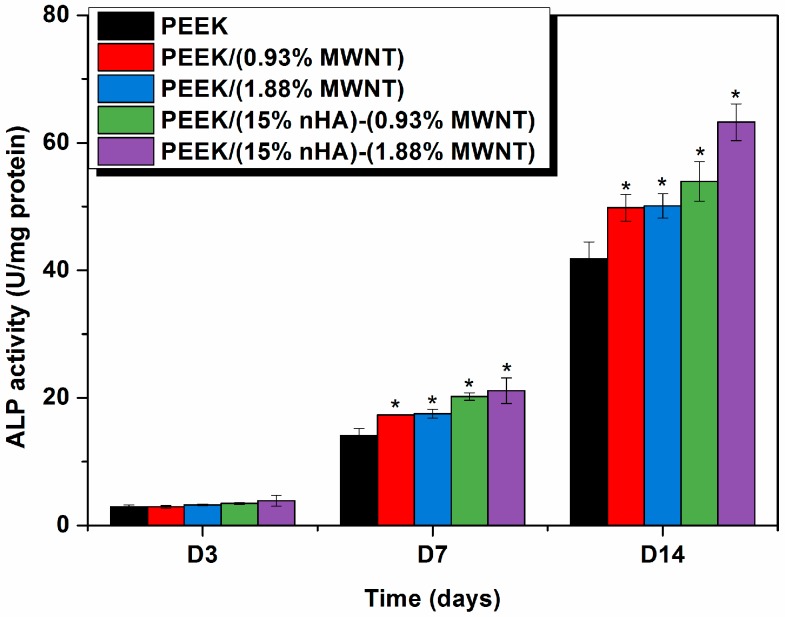
Alkaline phosphatase (ALP) activity normalized to protein content of murine MC3T3-E1 pre-osteoblasts cultured on PEEK and its nanocomposites for 1, 3 and 7 days. * represents *p* < 0.05.

Alkaline phosphatase (ALP) activity and calcium mineralization are mostly used as biochemical markers for early and late differentiation of osteoblast cells, respectively [49,50]. Bone formation involves a series of events that occur in the initial proliferation and differentiation stages, followed by mineralization. Proliferating osteoblasts show alkaline phosphatase activity in vitro; this is greatly enhanced during early differentiation of osteoblasts. Alkaline phosphatase catalyzes hydrolysis of *p-*nitrophenyl phosphate in alkaline buffer and produces phenol and inorganic phosphate. An enhanced level of this enzyme is required just before the onset of bone mineralization, providing localized enrichment of inorganic phosphate for nHA nucleation and proliferation. Figure 14 shows the ALP activity for the osteoblastic cells exposed to PEEK and its nanocomposites. Apparently, very low ALP activity is detected after 3 days for all samples investigated. At day 14, alkaline phosphatase activity of osteoblasts grown on the hybrids, especially PEEK/(15% nHA)-(1.88% MWNT) increases markedly in comparison to those observed in pure PEEK and PEEK-MWNT nanocomposites. This demonstrates the ability of this hybrid nanocomposite to enhance early osteoblast differentiation.

**Figure 15:**
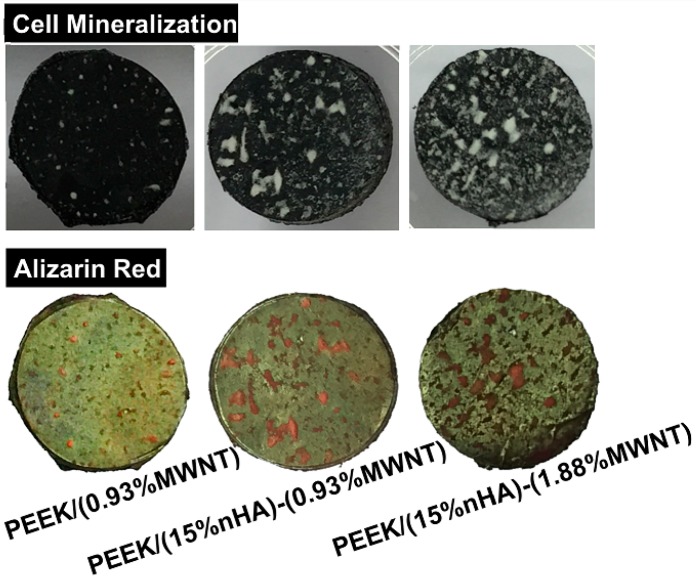
Representative photographs showing calcium mineralization (before staining) on PEEK/(0.93% MWNT), PEEK/(15% nHA)-(0.93% MWNT) and PEEK/(15% nHA)-(1.88% MWNT) nanocomposites cultured with murine MC3T3-E1 pre-osteoblasts for 28 days (**top**); Calcified nodules appeared as a dark red color on these samples after staining with Alizarin red-S (**bottom**).

**Figure 16:**
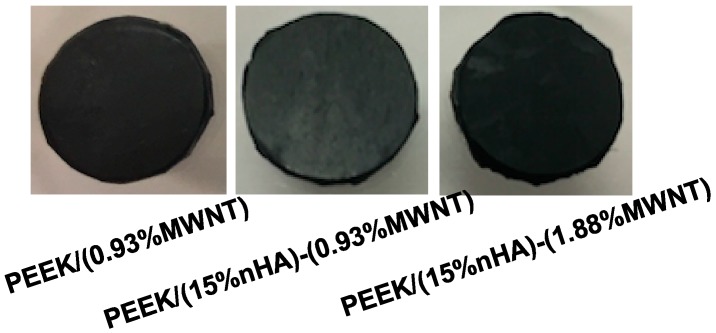
Photographs of Alizarin Red stained PEEK/(0.93% MWNT), PEEK/(15% nHA)-(0.93% MWNT) and PEEK/(15% nHA)-(1.88% MWNT) nanocomposites without cell exposure.

**Figure 17:**
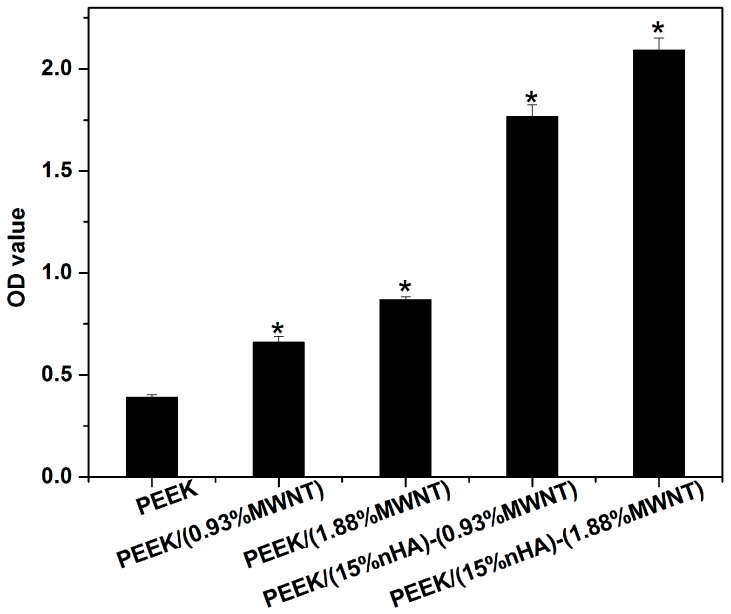
Optical absorbance of MC3T3-E1 pre-osteoblasts cultured on PEEK and its nanocomposites for 28 days and stained with Alizarin red. * represents *p* < 0.05.

Finally, Alizarin Red staining after 28 days of cell culture is used to determine the presence of calcium minerals on the PEEK-based nanocomposites. Bone forming cells containing calcium deposits are stained dark red by the Alizarin red solution. Prior to staining, white nodules can be seen on the PEEK/MWNT and PEEK/(15% nHA)-MWNT nanocomposites. However, large calcified nodules are more abundant in the hybrids (Figure 15). White mineralized nodules were stained a dark red color by alizarin red. Mineralized nodule formation indicates the final stages of osteoblastic differentiation at 28 days. To clarify that the nHA fillers of PEEK-based hybrids do not contribute to dark red staining, PEEK/(0.93% MWNT) composite and PEEK/(15% nHA)-MWNT hybrids without cells are also stained with Alizarin Red (Figure 16). Apparently, these specimens do not display dark red color, indicating that the nanohydroxyapatite in the hybrids does not contribute to the staining. Figure 17 shows the optical density (OD) of calcium mineralization of MC3T3-E1 pre-osteoblasts cultured on PEEK and its nanocomposites for 28 days. Low mineral deposition level occurs in pure PEEK as expected. Increased mineral deposition is found in the PEEK/(0.93% MWNT) and PEEK/(1.83% MWNT) nanocomposites comparing with neat PEEK. However, high mineralization level can be observed in both hybrids, especially in the PEEK/(15% nHA)-(1.88% MWNT) sample. Increased mineralization in cultured osteoblasts implies increased calcium deposition. As previously mentioned, the presence of nHA in PEEK-based hybrids converts hydrophobic PEEK to hydrophilic, thereby promoting protein adhesion, and subsequent osteoblastic cell attachment, proliferation, differentiation, and eventual calcium mineralization.

## 4. Conclusions

Melt-mixing and injection molding techniques have been successfully adapted for fabricating PEEK/(15% nHA)-MWNT hybrid nanocomposites. Combining the advantages of inorganic nHA and MWNTs, the resulting hybrid nanocomposites show good tensile property and excellent biocompatibility. In particular, the elastic modulus of PEEK can be increased from 3.87 to 7.13 GPa by adding 15 vol % nHA and 1.88 vol % MWNT. The tensile strength and elongation at break of the PEEK/(15% nHA)-(1.88% MWNT) hybrid are 64.48 MPa and 1.74%, respectively. Thus the tensile properties of this hybrid are superior to those of human cortical bone. Water contact angle measurements reveal that pure PEEK is hydrophobic with a water contact angle of 106°. Adding 1.88% MWNTs to PEEK decreases its water contact angles to 98°. The contact angle drops markedly to 77° by adding 1.88% MWNTs and 15% nHA hybrid fillers to PEEK. Accordingly, hydrophilic PEEK/(15% nHA)-(1.88% MWNT) hybrid favors the adhesion, proliferation and differentiation of osteoblasts on its surface based on the results of cell culture, fluorescence microscopy, MTT assay, WST-1 assay, ALP, and Alizarin red staining tests. Thus the PEEK/(15% nHA)-(1.88% MWNT) hybrid has the potential to be used for fabricating load-bearing orthopedic implants.

## Figures and Tables

**Table 1:** The compositions of binary PEEK/MWNT and ternary PEEK/(15 vol % nHA)-MWNT nanocomposites.

Sample	MWNT		nHA	
	vol %	wt %	vol %	wt %
PEEK	0	0	0	0
PEEK/(0.93% MWNT)	0.93	1.5	0	0
PEEK/(1.88% MWNT)	1.88	3.0	0	0
PEEK/(15% nHA)-(0.93% MWNT)	0.93	1.5	15	30
PEEK/(15% nHA)-(1.88% MWNT)	1.88	3.0	15	30

**Table 2:** Crystallization parameters of PEEK and its nanocomposites.

Sample	*T*_c_ (°C)	Δ*H*_c_ (J/g)	*X*_c_ (%)	FWHM (°C)
PEEK	281.14	59.38	45.68	7.57
PEEK/(0.93% MWNT)	285.91	59.26	46.28	8.81
PEEK/(1.88% MWNT)	286.58	59.85	47.46	9.62
PEEK/(15% nHA)-(0.93% MWNT)	284.90	36.87	41.40	9.21
PEEK/(15% nHA)-(1.88% MWNT)	284.73	35.97	41.30	9.07

**Table 3:** Tensile properties of PEEK and its nanocomposites.

Sample	Elastic modulus (GPa)	Tensile stress (Mpa)	Elongation (%)
PEEK	3.87 ± 0.10	80.06 ± 0.49	67.10 ± 13.40
PEEK/(0.93% MWNT)	4.21 ± 0.11	83.38 ± 0.78	57.25 ± 13.20
PEEK/(1.88% MWNT)	4.25 ± 0.85	82.08 ± 3.68	56.48 ± 24.90
PEEK/(15% nHA)-(0.93% MWNT)	6.83 ± 0.20	70.99 ± 7.51	2.32 ± 1.15
PEEK/(15% nHA)-(1.88% MWNT)	7.13 ± 0.12	64.48 ± 8.51	1.74 ± 0.58
PEEK/(15% nHA) [23]	6.20 ± 0.13	70.56 ± 3.22	2.71 ± 0.34
Human cortical bone [20]	7–30	50–150	1–3
